# Identification of Polyphenolics from *Loranthus globosus* as Potential Inhibitors of Cholinesterase and Oxidative Stress for Alzheimer's Disease Treatment

**DOI:** 10.1155/2021/9154406

**Published:** 2021-11-10

**Authors:** Netish Kumar Kundo, Md. Imran Nur Manik, Kushal Biswas, Riniara Khatun, Md. Yusuf Al-Amin, A. H. M. K. Alam, Toshihisa Tanaka, Golam Sadik

**Affiliations:** ^1^Department of Pharmacy, University of Rajshahi, Rajshahi 6205, Bangladesh; ^2^Department of Pharmacy, Mawlana Bhashani Science and Technology University, Tangail 1902, Bangladesh; ^3^Department of Pharmacy, Northern University, Dhaka 1205, Bangladesh; ^4^Department of Pharmacy, East West University, Dhaka 1212, Bangladesh; ^5^Department of Pharmacy, International Islamic University Chittagong, Chittagong 4318, Bangladesh; ^6^Department of Psychiatry, Osaka University Graduate School of Medicine, Osaka, Japan

## Abstract

Mistletoes are considered to be the potential medicinal herbs due to their rich traditional uses. *Loranthus globosus* is a Bangladeshi mango mistletoe that has been reported as folk medicine for various ailments and diseases. In an attempt to explore its effectiveness in Alzheimer's disease (AD), we investigated the antioxidant and acetylcholinesterase inhibitory activity of *L*. *globosus*. We report that the crude methanol extract (CME) of the plant contains a good amount of polyphenolics and possesses antioxidant and cholinesterase inhibitory activity. Fractionation of CME with solvents of varying polarity revealed the highest activity and polyphenolic content in the ethylacetate fraction (EAF). Correlation analysis revealed a significant (*P* < 0.05) association of polyphenolics with the antioxidant and cholinesterase inhibitory properties. Using column chromatography with diaion resin, the polyphenolics (EAF-PP) were isolated from the EAF that displayed the potent antioxidant and cholinesterase inhibitory activities. Kinetic analysis showed that EAF-PP exhibited a competitive type of inhibition. A total of thirty-six compounds including catechin and its different derivatives were identified in the EAF-PP by LC/MS analysis. Bioactivity-guided separation approach afforded the isolation of the two major active compounds catechin and catechin dimer from the EAF-PP. Hence, EAF-PP represents a potential source of antioxidants and cholinesterase inhibitors, which can be used in the management of AD.

## 1. Introduction

Among the neurodegenerative disorders, AD is the most devastative disorder of the aged people characterized by deficit of memory and cognition, psychobehavioral disturbances, and functional disabilities [1]. According to the recent estimate, approximately 36 million people are affected by AD around the world and it is projected that the number will be tripled by 2050 [2, 3]. Until now there is no effective treatment of this disorder. Therefore, exploration of new drugs for prevention of AD has become a priority now.

A common mechanism observed in the pathological process of neurodegenerative disorders including AD is oxidative stress, which originates due to disruption of balance between the oxidants and antioxidant system [4]. An increased production of reactive oxygen species (ROS), reactive nitrogen species (RNS), oxidation of lipid, protein, DNA and RNA, and glycoxidation have been observed in the brains of individuals with AD [5, 6]. The increased oxidative stress in the brain is largely due to the abundance of polyunsaturated fatty acids which are exposed to oxidation by free radicals or ROS. It is increasingly evident that Abeta protein, which is overproduced in AD, can generate ROS and free radicals and leads to peroxidation of lipid in the neuronal membrane [7]. This in turn disrupts the membrane integrity of the neuronal cells and causes cellular dysfunction [8]. Antioxidant therapies have been found to be effective in ameliorating the oxidative damage and improve the memory and cognitive function in the experimental model of AD [9]. In recent times, natural antioxidants have attracted intense interest because they are safe and display a diverse biological activity.

Current treatment of AD is mainly based on the restitution of the normal concentration of acetylcholine in the synaptic cleft through inhibition of acetylcholinesterase. Acetylcholine is a neurotransmitter secreted from the cholinergic neuron that plays a role in memory and cognition, which become deficient in AD [10]. According to cholinergic hypothesis, the deficiency of acetylcholine is correlated well with the cognitive decline and severity of dementia in AD [11]. Cholinesterases, acetylcholinesterase, and butyrylcholinesterase are the enzymes that catalyze the degradation of acetylcholine. Hence, inhibitors of acetylcholinesterase can increase the endogenous level of acetylcholine in the brain and improve cholinergic neurotransmission. Currently, four drugs have been licensed to treat AD; three of them are acetylcholinesterase inhibitors: donepezil, rivastimine, and galantamine [12]. These medications improve the symptoms for most patients with AD, but they are not able to completely stop or change the course of the illness [13]. Therefore, the researchers have been trying to develop an agent for treatment of AD that would target both the AChE and oxidative stress [14]. Medicinal plant has long been recognized as an alternative medicine and a rich source of novel drugs with potential therapeutic activity. Due to requirement of multitargeted agent for AD, plant has been the main focus for exploration of drugs as it contains a large number of biosynthetic intermediates that show specificities for different targets [15].

Mistletoes are considered as all-purpose herbs due to their rich traditional uses. *Loranthus globosus* (locally called Chota Manda), a Bangladeshi mistletoe of the family *Loranthaceae*, has been used as folk medicine for different diseases. The extract of this plant is used traditionally to treat women's diseases, particularly the menstrual trouble and to check abortion [16]. The bark of this plant has astringent and diuretic properties and used in the treatment of chronic wounds, hepatic disorder, disease of the spleen, inflammation, hypertension, allergy, and itching. An infusion of the bark and leaves is used to treat acute and chronic diarrhea, and in combination with other plant, it is used in skin infection [17]. We have previously reported the antimicrobial activity and cytotoxicity of the extracts of *L. globosus* barks and characterized several important bioactive compounds including catechin, 3,4-dihydroxy-cinnamyl alcohol and 2,3,4-trihydroxy-cinnamyl alcohol [18, 19]. These metabolites have shown earlier the antioxidant and neuroprotective effects [20]. Literature review of the different species of Loranthus revealed that they have potential antioxidant activity due to rich polyphenolic constituents [21–27]. Although *L. globosus* has potential medicinal values, no works have been carried out yet for its effectiveness in AD. In this study, we have investigated the antioxidant and cholinesterase inhibitory activities of the extracts of *L. globosus* and to explore the compounds responsible for activity.

## 2. Materials and Methods

### 2.1. Chemicals

2,2′-Diphenyl-1-picrylhydrazyl (DPPH), 5,5′-dithio-bis-(2-nitro) benzoic acid (DTNB), thiobarbituric acid (TBA), 2-deoxy-D-ribose, acetylthiocholine, S-butyrylthiocholine, donepezil, and galantamine were procured from Sigma-Aldrich, Germany. silica gel GF_264_, silica gel 60-120, Folin-Ciocalteu reagent, Tris-HCl, aluminum chloride, potassium ferricyanide, ammonium molybdate, trichloroacetic acid (TCA), and triton X-100 were from Merck, Darmstadt, Germany. Catechin, gallic acid, and ascorbic acid were purchased from Wako Pure Chemical Company Ltd., Osaka, Japan. Petroleum ether, chloroform, ethylacetate, and methanol were obtained from Active Fine Chemicals Limited, Dhaka, Bangladesh. Unless specified, all other chemicals were of analytical grade.

### 2.2. Collection and Extraction of Plant Materials

The fresh barks of the plant *L. globosus* were collected from the campus of Rajshahi University in February 2017. The plant was authenticated by an expert of the Department of Botany, Rajshahi University, and a voucher specimen (accession no. 98) has been deposited at the herbarium of the department.

The plant material was washed with distilled water, cut into small pieces, shade dried, and ground to coarse powder by grinding machine. The powder (1.5 kg) was immersed in methanol for several days with occasional stirring. It was then filtered and concentrated *in vacuo* with a rotary evaporator to yield the semisolid mass The crude methanolic extract (CME, 61.8 g) was then suspended in 10% methanol and successively partitioned with petroleum ether (PEF), chloroform (CLF), ethylacetate (EAF), and water (AQF) using separating funnel as described earlier [28]. The yield corresponding extracts were 5.3 g, 8.5 g, 28 g, and 20 g, respectively. They were stored at 4°C in a refrigerator until further use.

### 2.3. Phytochemical Analysis

#### 2.3.1. Total Phenolic Content (TPC)

The TPC of the extract and fractions from *L. globosus* was estimated by the Folin-Ciocalteu method as described by Singleton [29]. Briefly, the extract/fraction (0.5 ml) was added to 10% Folin-Ciocalteu reagent (2.5 ml) and 7.5% sodium carbonate solution (2.5 ml) and left in the dark for 20 minutes. The absorbance was read at 760 nm by a spectrophotometer. As the standard phenolics, gallic acid was used. The phenolic content was obtained from the standard curve for gallic acid.

#### 2.3.2. Quantitation of Total Flavonoid Content (TFC)

The TFC was determined by the aluminum chloride method [30]. Briefly, the extract/fraction (1.0 ml) was added to methanol (3.0 ml), 10% AlCl_3_ (0.2 ml), 1 M potassium acetate (0.2 ml), and distilled water (5.6 ml) and left at room temperature for 30 minutes. The absorbance was read at 420 nm by a spectrophotometer. As the standard flavonoid, catechin was used. The flavonoid content was calculated from the standard curve for catechin.

#### 2.3.3. Quantitation of Total Proanthocyanidin Content (TPAC)

The TPAC was measured by the vanillin/HCl assay according to the method of Sun et al. [31]. Briefly, the extract/fraction (0.5 ml) was added to a 4% vanillin-methanol solution (3 ml) and hydrochloric acid (1.5 ml). The mixture was left for 15 min at room temperature, and then, the absorbance was read at 500 nm. Total proanthocyanidin content was calculated from the standard curve for catechin.

### 2.4. Antioxidant Activity

#### 2.4.1. Reducing Power

Reducing power of the extract/fractions was determined following the method of Oyazu [32]. Sample at various concentrations was added to 0.2 M potassium buffer (2.5 ml) and 1% potassium ferricyanide (2.5 ml) followed by incubation at 50°C for 20 minutes. Following incubation, TCA solution (10%, 2.5 ml) was added in the reaction mixture and centrifuged at 3000 rpm for 10 minutes. The resulting supernatant (2.5 ml) was mixed with equal volume of water (2.5 ml) and 0.1% ferric chloride solution (0.5 ml). The absorbance of the solution was read at 700 nm by a spectrophotometer. As a positive control, standard antioxidant catechin was used.

#### 2.4.2. Total Antioxidant Capacity Assay

Total antioxidant activity was estimated according to the method of Prieto et al. [33]. Briefly, the extract/fractions were added to sodium phosphate (28 mM), sulphuric acid (0.6 M), and ammonium molybdate (4 mM) followed by heating at 95°C for 90 min. It was allowed to cool, and then, the absorbance was read at 695 nm. Catechin was used as positive control.

#### 2.4.3. DPPH Radical Scavenging Assay

DPPH radical scavenging activity of the extract/fractions was determined by the method of Choi et al. [34]. Briefly, sample in methanol at various concentrations (6.25-100 *μ*g/ml) was added to DPPH (0.135 mM) and left in the dark at room temperature for 30 minutes. Then, the absorbance was read at 517 nm by a spectrophotometer. Catechin was used as positive control. The following equation was used to calculate the percent scavenging:

(1) $$\begin{array}{c} \left[\frac{\left(A_{\text{control}}–A_{\text{sample}}\right)}{A_{\text{control}}}\right]\times 100, \end{array}$$

where *A*_control_ is the absorbance of control and *A*_sample_ is the absorbance of sample. IC_50_ values were obtained from the plot of the percentage inhibition against the compound concentration.

#### 2.4.4. Hydroxyl Radical Scavenging Activity

Hydroxyl radical scavenging activity of the extract/fractions was estimated by the method as described earlier [35]. Sample at various concentrations was added to 1 ml reaction mixture containing 2.8 mM 2-deoxy-2-ribose, 20 mM phosphate buffer pH 7.4, 100 *μ*M EDTA, 1 mM H_2_O_2_, 100 *μ*M FeCl_3_, and 100 *μ*M ascorbic acid and then incubated for 1 h at 37°C. To the reaction mixture (0.5 ml), TCA (1 ml, 2.8%) and TBA (1 ml, 1%) were added and heated at 90°C for 15 minutes followed by cooling. The absorbance of the solution was read at 532 nm by a spectrophotometer against an appropriate blank solution. Catechin was used as a positive control. Similar to DPPH assay, the percent scavenging of hydroxyl radical was calculated. IC_50_ values were obtained from the plot of the percentage inhibition against the compound concentration.

#### 2.4.5. Lipid Peroxidation Inhibitory Activity

Lipid peroxidation inhibitory activity of the extract and fractions was determined by the TBA method as described by Liu et al. [36]. Brain homogenate was used as the source of lipid which was prepared from mice by the method as described earlier [37]. In brief, brain was homogenized in 50 mM phosphate buffer (pH 7.4) and centrifuged at 10000 g at 4°C for 20 min to yield the supernatant. Sample at various concentrations was added to the brain homogenates (0.5 ml), 10 *μ*M hydrogen peroxide (100 *μ*l), and 0.15 M KCl (1 ml) and incubated for half an hour at 37°C. Following incubation, 15% TCA, 0.38 TBA, and 5% BHT in 2 ml of HCl (0.25 N) were mixed with the reaction mixture and heated at 80°C for 60 minutes followed by cooling. The resulting mixture was centrifuged to separate the supernatant, and the absorbance was measured at 532 nm by a spectrophotometer. As positive control catechin was used. Similar to DPPH assay, the percent inhibition of lipid peroxidation was calculated. IC_50_ values were obtained from the plot of the percentage inhibition against the compound concentration.

### 2.5. Cholinesterase Inhibitory Activities

The widely used Ellman method was used to determine the ability of the extract/fraction to inhibit acetylcholinesterase (AChE) and butyrylcholinesterase (BChE) [38]. Mouse brain AChE enzyme and blood BChE enzyme were prepared by the method as described earlier [37]. For AChE inhibition, acetylthiocholine iodide (ATCI) was used as substrate, and for BChE inhibition, S-butyrylthiocholine iodide (BTCI) was used. The enzymatic hydrolysis of ATCI and BTCI was monitored by following the formation of yellow 5-thio-2-nitrobenzoate anion at 412 nm using a spectrophotometer. Sample at various concentrations was incubated with enzyme solution at 37°C for 15 min to allow for inhibition and then mixed with and DTNB (1 mM) in sodium phosphate buffer (50 mM, pH 8.0). The reaction was initiated by addition of ATCI (0.5 mM), and the absorbance of the mixture was determined against a control solution. The analyses were performed in triplicate. Donepezil was used as reference AChE inhibitor, and galantamine was used as reference BChE inhibitor. The following equation was used to calculate the percent inhibition of cholinesterase activity:

(2) $$\begin{array}{c} \left[\frac{\left(A_{\text{control}}–A_{\text{sample}}\right)}{A_{\text{control}}}\right]\times 100, \end{array}$$

where *A*_control_ is the absorbance of control and *A*_sample_ is the absorbance of extract or fractions. The dose response curve obtained by plotting the percent inhibition values against test concentrations was used to calculate IC_50_ values of each extract/fraction and compounds.

### 2.6. Isolation of Polyphenols from EAF and AQF

For isolation of polyphenolics, EAF and AQF were subjected to column chromatography separately with Diaion HP 20 resin as stationery phase followed by elution with methanol as the mobile phase according to the manufacturer's instruction. The polyphenolics from EAF (EAF-PP) and AQF (AQF-PP) were freeze-dried and stored at 4°C until use.

### 2.7. Inhibition Kinetics of Cholinesterase Enzymes by EAF-PP

The kinetic mode of AChE and BChE inhibition by EAF-PP was determined by preparing a range of EAF-PP concentrations (100, 200, and 400 *μ*g/mL) in which the concentration of the substrate (ATCI/BTCI) was varied (1.4, 0.7, 0.35, 0.175, and 0.0875 mM). With different concentration (S) of substrate (ATCI/BTCI), the velocity of the enzyme inhibition was different. The assay was carried in triplicate. Lineweaver-Burk graph was plotted from *S*^−1^ vs. *V*^−1^ to determine the type of inhibition [39]. From these data, *V*_max_ (maximum reaction velocity) and *K*_*m*_ (dissociation constant) were calculated.

### 2.8. LC-MS Analysis of EAF-PP

The mass spectrum of the compounds in the EAF-PP was analyzed by LC-MS (Shimadzu 8050, Shimadzu, Japan) system. For separation of compounds, the sample (10 ml) was subjected to a C18 column (100 Å, 5 *μ*m, and 2.1 mm × 150 mm) with a gradient mobile phase consisting of water (A) and methanol (B) containing 0.1% formic acid at a flow rate of 0.5 ml/min. The mass spectra were recorded on with positive and negative ionization mode. The operation was carried out with the following conditions: interface temperature 300°C, the interface voltage 4.5 kV for positive mode, and -3.5 kV for negative mode; nebulizer gas N2, 3 L/h; drying gas 10 L/h; and desolvation line temperature 15°C and heat block temperature 500°C. The compounds were identified by comparing their *m*/*z* ratio with those published in the literature. The data acquisition was performed, scanning from values of *m*/*z* 100 to 1800 [40].

### 2.9. Purification and Characterization of Major Active Compounds from the EAF-PP

The active compounds in the EAF-PP were isolated and purified by a combination of column chromatography (CC) and preparative thin layer chromatography (PTLC). The EAF-PP was chromatographed on an open column with silica gel 60 (Merck, Germany) as a stationary phase followed by elution with gradient system consisting of n-hexane, chloroform, and methanol. The fractions were monitored on TLC, visualized under UV light, and combined based on similar *R*_*f*_ values. Seven major subfractions (F1 to F7) were obtained, and among them, F1 and F2 showed high antioxidant and cholinesterase inhibitory activity. F1 appeared as a single spot representing a single compound C-**1** (54 mg), whereas the compound **2** (18 mg) was purified from the F2 was purified by preparative thin layer chromatography on silica gel GF_254_ with n-hexane-acetone (6 : 4) as the mobile phase.

The compound **1** was characterized as catechin by comparing its *R*_*f*_ value with those of an authentic sample, while the compound **2** was characterized by nuclear magnetic resonance (NMR) spectroscopy. The compound was dissolved in deuterated methanol and subjected to a Jeol-Ex 400 MHz spectrometer for ^1^H- and FT-NMR 100 MHz spectrometer for ^13^C-NMR spectra. The structure of the compound **2** was confirmed by comparing its spectral data with the reported values in the literature [23, 41].

### 2.10. Statistical Analysis

All experiments were done in triplicate, and the results were reported as mean ± SD. Graph Pad Prism (version 8.0.1) and Microsoft Excel 2010 were used for the statistical and graphical evaluations. The statistical significance (*P* value <0.05) between the means was calculated using one-way analysis of variance (ANOVA). Correlation study was performed using Pearson correlation test.

## 3. Results

### 3.1. Phytochemical Analysis

Quantitative analysis of the CME of *L. globosus* and its fractions was carried out for the total content of phenolics, flavonoids, and proanthocyanidin, and the results have been presented in Table 1. The results demonstrated that the CME possesses a good amount of phenolics (336.989 ± 1.837 mg GAE/g dried extract), flavonoids (180.00 ± 2.06 mg GAE/g dried extract), and proanthocyanidin (291.00 ± 1.50 mg GAE/g dried extract). Following fractionation of the CME, the highest content was found in the EAF followed by AQF, PEF, and CLF. The total phenolic contents of EAF, AQF, PEF, and CLF were 270.466 ± 0.657, 240.932 ± 1.51, 64.803 ± 0.448, and 30.753 ± 1.55 mg GAE/g dried extract, while the flavonoid contents were 281.715 ± 2.06, 60.191 ± 3.805, 49.714 ± 1.512, and 5.267 ± 0.162 mg GAE/g dried extract, and that of the total proanthocyanidin contents were 278.00 ± 2.291, 179.00 ± 0.866, 57.00 ± 1.50, and 21.00 ± 1.50 mg CE/g dried extract, respectively.

### 3.2. Antioxidant Activity

**Figure 1:**
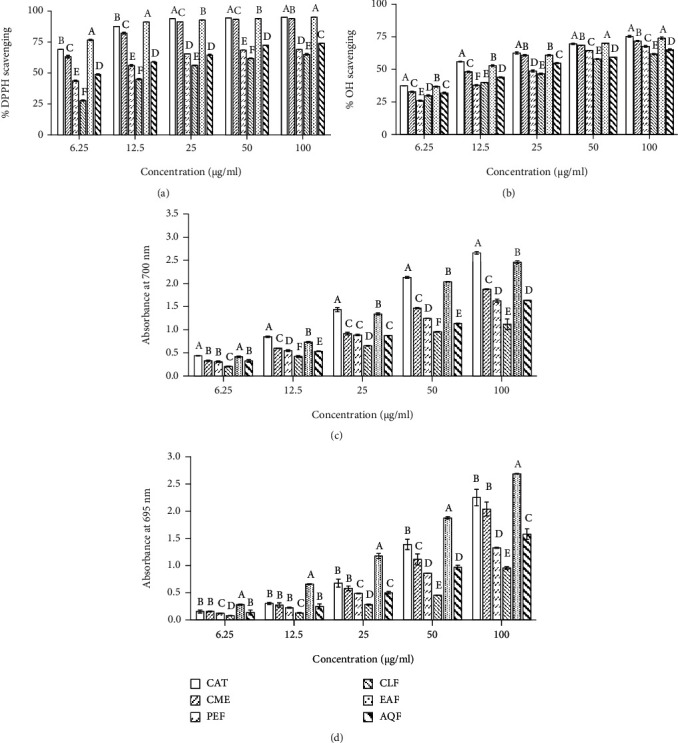
Antioxidant activities of the extract and fractions from *L. globosus*. (a) DPPH radical scavenging activity. IC_50_ (*μ*g/ml): CME, 4.156 ± 0.088; PEF, 11.223 ± 0.248; CLF, 24.617 ± 0.421; EAF, 3.130 ± 0.022; AQF, 7.975 ± 0.225; CAT, 3.41 ± 0.004. (b) Hydroxyl radical scavenging activity. IC_50_ (*μ*g/ml): CME, 15.60 ± 0.356; PEF, 26.617 ± 0.293; CLF, 31.697 ± 0.570; EAF, 12.623 ± 0.268; AQF, 22.687 ± 0.389; CAT, 11.333 ± 0.356. (c) Reducing power. At 100 *μ*g/ml concentration, the absorbances are as follows: CME, 1.874 ± 0.014; PEF, 1.624 ± 0.036; CLF, 1.117 ± 0.116; EAF, 2.457 ± 0.034; AQF, 1.634 ± 0.006; CAT, 2.660 ± 0.032. (d) Total antioxidant capacity. At 100 *μ*g/ml concentration, the absorbances are as follows: CME, 2.039 ± 0.129; PEF, 1.326 ± 0.009; CLF, 0.954 ± 0.025; EAF, 2.688 ± 0.008; AQF, 1.578 ± 0.098; CAT, 2.251 ± 0.151. Results are expressed as mean ± SD (*n* = 3). Means with different letters (a-f) differ significantly (*P* < 0.05). CME: crude methanolic extract; PEF: petroleum ether fraction; CLF: chloroform fraction; EAF: ethylacetate fraction; AQF: aqueous fraction; CAT: catechin.

We evaluated the antioxidative property of the CME of *L. globosus* and its fractions using several *in vitro* models. DPPH and hydroxyl free radicals scavenging models were used to assess the capacity of the extract/fractions to scavenge the free radicals, and the results have been shown in Figures 1(a) and 1(b). In both assays, the CME showed marked activity as judged by their IC_50_ values. The values were 4.156 ± 0.088 *μ*g/ml and 15.60 ± 0.375 *μ*g/ml for DPPH and hydroxyl radical scavenging, respectively. Under the same condition, the IC_50_ values of the reference standard catechin were 3.41 ± 0.004 *μ*g/ml and 11.333 ± 0.356 *μ*g/ml, respectively. When CME was fractionated, the activity was found to be distributed in all the fractions. However, the activity was high in the EAF followed by AQF, PEF, and CLF. Their IC_50_ values were 3.130 ± 0.022, 7.975 ± 0.225, 11.223 ± 0.248, and 24.617 ± 0.421 *μ*g/ml for DPPH radical scavenging and 12.623 ± 0.268, 22.687 ± 0.389, 26.617 ± 0.293, and 31.697 ± 0.570 *μ*g/ml for hydroxyl scavenging, respectively. We have noted high DPPH scavenging activity of EAF than that of the standard antioxidant catechin.

The antioxidant activity of the extract/fractions was further evaluated by reducing power and total antioxidant capacity assays which reflected their capacity to donate electron or proton. In reducing power assay, the crude extract exhibited good reducing activity and the activity was increased in a dose-dependent manner (Figure 1(c)). The absorbance of CME was 1.874 ± 0.014 at a concentration of 100 *μ*g/ml. Among the fractions, EAF showed the highest activity and CLF, the lowest. The absorbance of EAF, AQF, PEF, and CLF was 2.457 ± 0.034, 1.634 ± 0.006, 1.624 ± 0.036, and 1.117 ± 0.116, respectively, at the same concentration. In the total antioxidant activity assay based on the capacity to reduce Mo (VI) to Mo (V), the CME exhibited good activity with absorbance of 2.039 ± 0.129 at 100 *μ*g/ml concentration (Figure 1(d)). Similar to reducing power, the highest total antioxidant activity was found in the EAF followed by AQF, PEF, and CLF with the absorbance of 2.688 ± 0.008, 1.578 ± 0.098, 1.326 ± 0.009, and 0.954 ± 0.025, respectively. Importantly, in this assay, the EAF showed more activity than that of the antioxidant catechin.

**Figure 2:**
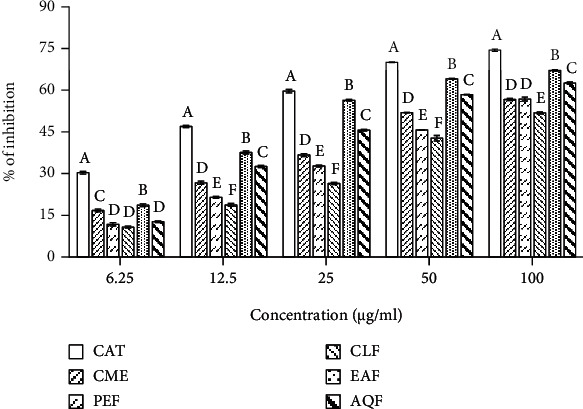
Lipid peroxidation inhibitory activity of the extract and fractions from *L. globosus*. IC_50_ (*μ*g/ml): CME, 56.073 ± 1.176; PEF, 66.003 ± 1.754; CLF, 85.863 ± 0.246; EAF, 25.997 ± 0.246; AQF, 38.087 ± 0.417; CAT, 16.510 ± 0.123. Results are expressed as mean ± SD (*n* = 3). Means with different letters (a-f) differ significantly (*P* < 0.05). CME: crude methanolic extract; PEF: petroleum ether fraction; CLF: chloroform fraction; EAF: ethylacetate fraction; AQF: aqueous fraction; CAT: catechin.

Free radicals are reported to directly attack lipid, resulting in lipid peroxidation [42]. We evaluated the potential of the CME and its fractions to inhibit the peroxidation of lipid by the TBA method, and the results have been shown in Figure 2. An increased peroxidation of lipid was observed in the brain homogenates in the presence of hydrogen peroxide which was indicated by formation of pink color, and the CME showed considerable inhibition of lipid peroxidation with IC_50_ value of 56.073 ± 1.176 *μ*g/ml. EAF exhibited the highest activity among the fractions followed by AQF, PEF, and CLF with IC_50_ values of 25.997 ± 0.246, 38.087 ± 0.417 *μ*g/ml, 66.003 ± 1.754, and 85.863 ± 0.246 *μ*g/ml, respectively. The high activity of EAF and AQF indicated that they might be effective in the inhibition of lipid caused by free radicals.

### 3.3. Cholinesterase Inhibitory Activity

**Figure 3:**
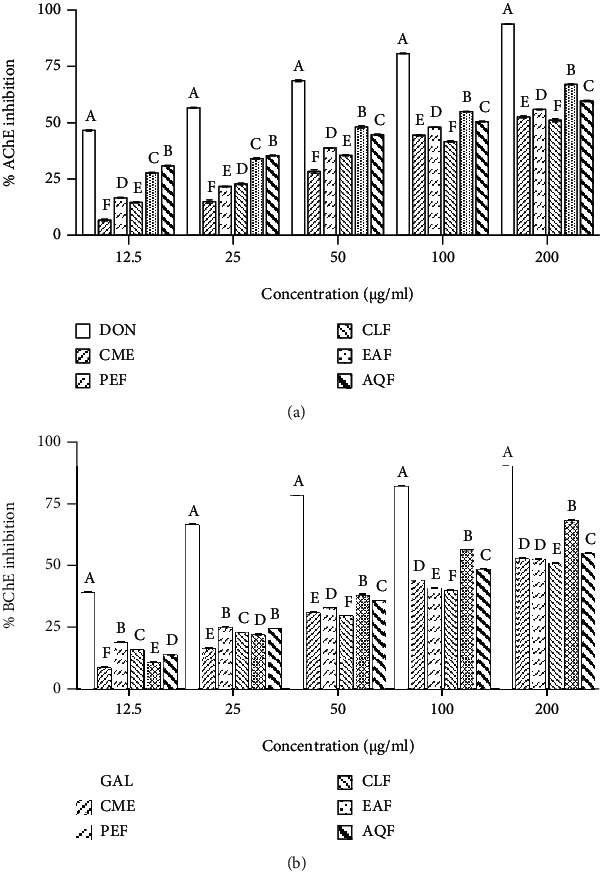
Cholinesterase inhibitory activities of the extract and fractions from *L. globosus*. (a) Inhibition of AChE. IC_50_ (*μ*g/ml): CME, 153.767 ± 2.409; PEF, 123.367 ± 0.306; CLF, 171.533 ± 5.478; EAF, 64.987 ± 0.669; AQF, 87.417 ± 0.610; DON, 8.351 ± 0.076. (b) Inhibition of BChE. IC_50_ (*μ*g/ml): CME, 155.733 ± 0.907; PEF, 353.633 ± 3.408; CLF, 391.633 ± 4.561; EAF, 85.270 ± 0.982; AQF, 129.267 ± 1.002; GAL, 8.208 ± 0.105. Results are expressed as mean ± SD (*n* = 3). Means with different letters (a-f) differ significantly (*P* < 0.05). CME: crude methanolic extract; PEF: petroleum ether fraction; CLF: chloroform fraction; EAF: ethylacetate fraction; AQF: aqueous fraction; AChE: acetylcholinesterase; BChE: butyrylcholinesterase; DON: donepezil; GAL: galantamine.

Inhibition of acetylcholinesterase (AChE) and butyrylcholinesterase (BChE) by the CME and its fractions were evaluated by the Ellman's method [38], and the results have been shown in Figure 3. The CME exerted inhibition of both AChE and BChE in a dose-dependent manner, with IC_50_ values of 153.767 ± 2.409 and 155.733 ± 0.907 *μ*g/ml, respectively (Figures 3(a) and 3(b)). Of the fractions, highest activity was found in the EAF followed by AQF, CLF, and PEF with IC_50_ values of 64.987 ± 0.669, 87.417 ± 0.610, 171.533 ± 5.478, and 123.367 ± 0.306 *μ*g/ml for AChE and 85.270 ± 0.982, 129.267 ± 1.002, 391.633 ± 4.561, and 353.633 ± 3.408 *μ*g/ml for BChE, respectively. Under the same condition, the reference AChE inhibitor donepezil showed an IC_50_ of 8.351 ± 0.076 *μ*g/ml and the reference BChE inhibitor galantamine had an IC_50_ of 8.208 ± 0.105 *μ*g/ml. Taken together, the EAF and AQF display appreciable inhibition against both AChE and BChE enzymes.

### 3.4. Correlation between Total Phenolic, Flavonoid, and Proanthocyanidin Contents and the Antioxidant and Cholinesterase Inhibitory Activities

Since the antioxidant and cholinesterase inhibitory activity was found to be high in the fractions which are rich in polyphenolics, we hypothesized that the polyphenolics might be associated with the activity. To investigate the correlation of phenolics, flavonoids, and proanthocyanidins with the antioxidant and cholinesterase inhibitory activities, the correlation studies were performed and the results have been given in Table 2. It has been observed that the total flavonoid content is significantly correlated with DPPH radical scavenging activity (*R*^2^ = 0.6021, *P* < 0.05), hydroxyl radical scavenging (*R*^2^ = 0.8771, *P* < 0.01), reducing activity (*R*^2^ = 0.881, *P* < 0.01), total antioxidant activity (*R*^2^ = 0.9267, *P* < 0.001), lipid peroxidation inhibition (*R*^2^ = 0.693, *P* < 0.05), AChE (*R*^2^ = 0.6274, *P* < 0.05), and BChE (*R*^2^ = 0.741, *P* < 0.05) inhibition. The phenolic content showed a significant correlation with DPPH radical scavenging activity (*R*^2^ = 0.7689, *P* < 0.01), hydroxyl radical scavenging (*R*^2^ = 0.8692, *P* < 0.01), reducing activity (*R*^2^ = 0.6179, *P* < 0.05), total antioxidant activity (*R*^2^ = 0.7653, *P* < 0.01), lipid peroxidation inhibition (*R*^2^ = 0.6976, *P* < 0.05), and BChE (*R*^2^ = 0.9056, *P* < 0.001) inhibition. The total proanthocyanidin content was correlated significantly with DPPH radical scavenging activity (*R*^2^ = 0.6612, *P* < 0.05), hydroxyl radical scavenging (*R*^2^ = 0.9015, *P* < 0.01), total antioxidant activity (*R*^2^ = 0.8665, *P* < 0.01), lipid peroxidation inhibition (*R*^2^ = 0.681, *P* < 0.05), and BChE (*R*^2^ = 0.8274, *P* < 0.01) inhibitory activities. The significant correlation found between polyphenolics and antioxidant and cholinesterase inhibitory activities indicated that the polyphenolics might be involved in the bioactivities.

### 3.5. Isolation of Polyphenolics from EAF and AQF and Determination of Their Activity

**Figure 4:**
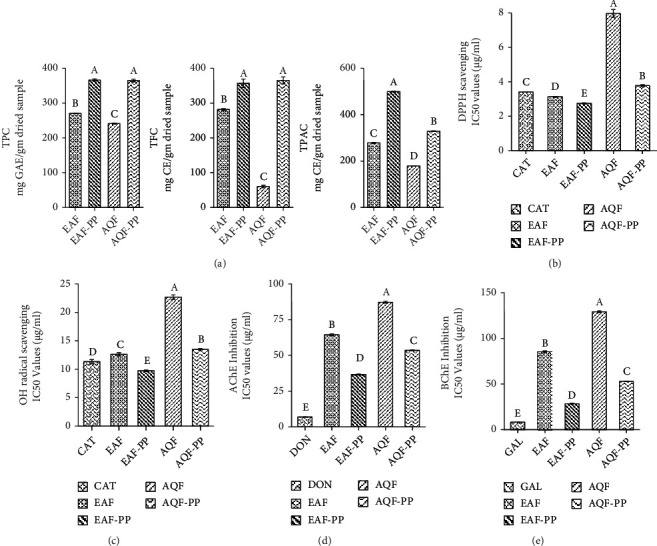
Quantitative analysis of the EAF-PP and AQF-PP and assessment of their activities. (a) Total phenolic, total flavonoid, and total proanthocyanidin contents. (b) DPPH scavenging activity. (c) Hydroxyl scavenging activity. (d) AChE inhibitory activity. (e) BChE inhibitory activity. Activities were expressed as IC_50_. Data represent as mean ± SD (*n* = 3). Means with different letters (a-f) differ significantly (*P* < 0.05). EAF: ethylacetate fraction; AQF: aqueous fraction; CAT: catechin; TPC: total phenolic content; TFC: total flavonoid content; TPAC: total proanthocyanidin content; GAE: gallic acid equivalent; CE: catechin equivalent; AChE: acetylcholinesterase; BChE: butyrylcholinesterase; DON: donepezil; GAL: galantamine; EAF-PP: polyphenols from EAF; AQF-PP: polyphenols from AQF.

To further understand the role of polyphenolics, the polyphenolics EAF-PP and AQF-PP were isolated from the EAF and AQF separately by using column chromatography with diaion resin and assessed for their activities. As shown in Figure 4, EAF-PP and AQF-PP were found to possess more phenolics (366.380 ± 3.119 and 364.731 ± 3.983 mg GAE/g extract), flavonoids (357.143 ± 11.999 and 365.143 ± 10.286 mg CE/g extract), and proanthocyanidins (499.5 ± 1.5 and 328.5 ± 1.5 mg CE/g extract) and exhibited potent antioxidant and cholinesterase inhibitory activity. The IC_50_ values of EAF-PP and AQF-PP were 37.617 ± 0.323 and 54.283 ± 0.289 *μ*g/ml for AChE inhibition and 28.42 ± 0.404 *μ*g/ml and 2.843 ± 0.123 *μ*g/ml for BChE inhibition, respectively, while the values were 2.747 ± 0.026 and 3.777 ± 0.055 *μ*g/ml, for DPPH free radical scavenging, and 9.736 ± 0.116 and 13.483 ± 0.150 *μ*g/ml for hydroxyl free radical scavenging, respectively. Comparing with AQF-PP, EAF-PP was more potent in terms of antioxidant and cholinesterase inhibitory activities.

### 3.6. Analysis of Mode of Inhibition of EAF-PP

**Figure 5:**
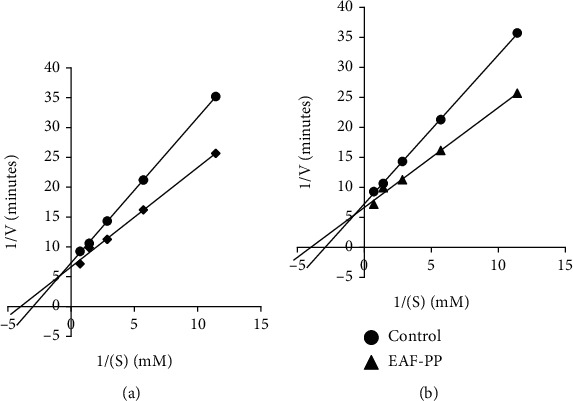
Lineweaver-Burk plot for inhibition of (a) AChE and (b) BChE by different concentrations of EAF-PP. Results represent the average values (*n* = 3). AChE: acetylcholinesterase; BChE: butyrylcholinesterase.

Since EAF-PP showed strong inhibition against both the AChE and BuChE, we investigated further to determine the modes of enzyme inhibition of this fraction using Lineweaver-Burke plots. Plots of AChE and BuChE inhibition by EAF-PP were linear and intersected at a point on *x*-axis (Figure 5). These results indicated that the EAF-PP is a noncompetitive inhibitor for both the AChE and BChE enzymes.

### 3.7. LC-MS Analysis of the EAF-PP

LC/MS is an important technique for qualitative analysis of the phytochemicals in the extract. To identify the polyphenolic compounds that are contributing to the bioactivity, the EAF-PP was analyzed by LC-MS and the compounds' profile has been shown in Table 3 [24, 43–53]. Thirty-six compounds were tentatively assigned on the basis of *m*/*z* comprising of phenolic acids, flavonoids, and proanthocyanidins. The identified phenolic acids were gallic acid and its derivatives (ethyl gallate, gallic acid 3-*O*-gallate, galloyl glucose, and octa-*O*-galloyl glucose), *p*-coumaric acid and its derivatives (p-coumaroyl tartaric acid and *p*-coumaroyl-4-*O*-glucoside), quinic acid, ferulic acid, coniferin, *p*-amino benzoate, syringetin-7-*O*-hexoside, rosamarinic acid, sinapic acid hexoside, and linamarin gallate. The flavonoids that were detected include catechin and its derivatives (catechin-3-*O*-gallate, epicatechin 3,5,7-gallate, epigallocatechin 3-*O*-gallate, epigallocatechin dimer, and *O*-methylated (+) catechin gallate), kaempferol and its derivatives (kaemferol-3-*O*-acetylglucoside and kaemferol-7-*O*-rhamnoside), apigenin and its derivative apigenin-7-*O*-glucoside, quercetin and its derivative 3,7-Di-*O*-methyl quercetin, naringenin and naringin-7-*O*-glucoside, rhamnetin, and myrecetin rhamnohexoside. Catechin dimer and catechin tetramer glucose appeared to be the proanthocyanidin.

### 3.8. Activity-Guided Isolation of Active Compound from EAF-PP

**Figure 6:**
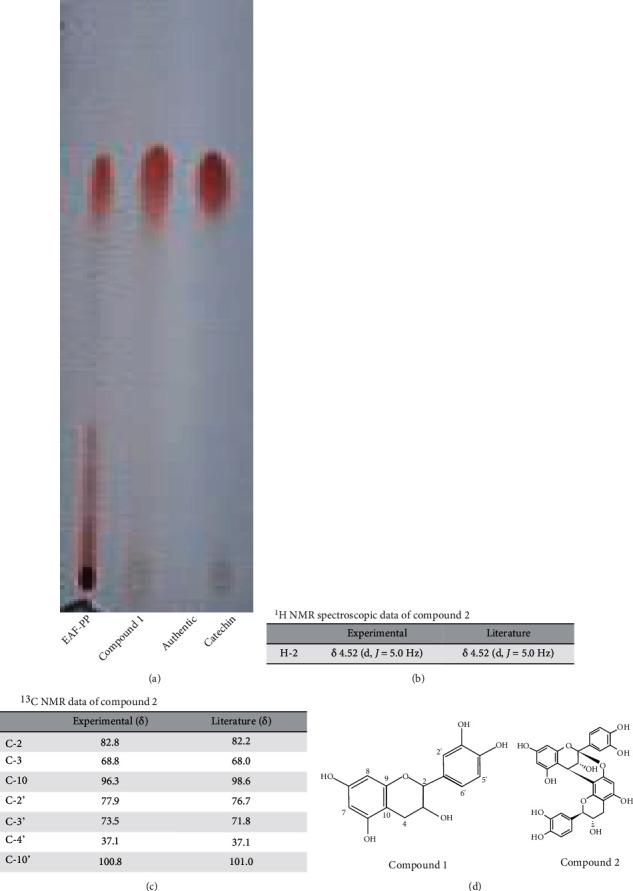
Characterization of the compounds isolated from the polyphenolic EAF-PP. (a) TLC profile of the compound **1** and authentic catechin. (b) ^1^H NMR spectroscopic data of the compound **2**. (c) ^13^C NMR spectroscopic of the compound **2**. (d) Chemical structures of the compounds **1** and **2**.

**Figure 7:**
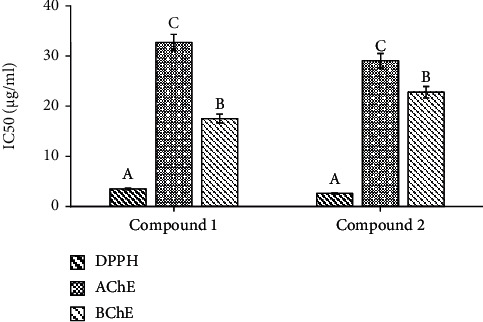
Antioxidant and cholinesterase inhibitory activities of the compounds **1** and **2**. IC_50_ values of the compounds for DPPH radical scavenging and inhibition of AChE and BChE were shown. Results are expressed as mean ± SD (*n* = 3). Means with different letters (a-c) differ significantly (*P* < 0.05). DPPH: 2,2-diphenyl-1-picrylhydrazyl; AChE: acetylcholinesterase; BChE: butyrylcholinesterase.

Initial identity of the compounds present in the EAF-PP prompted us to define the role of compounds in the bioactivity. We followed the activity-guided chromatographic separation approach that afforded the isolation of two major compounds **1** and **2** from the EAF-PP (Figure 6). The compound **1** was identified as catechin by direct comparison of its *R*_*f*_ value (0.6; chloroform : ethylacetate, 1 : 2.5) with that of the authentic sample as well as the previously reported compound from this plant (Figure 6(a)) [18], while the compound **2** was characterized as catechin dimer by comparison of its ^1^H- and ^13^C-NMR spectral data with the values published in the literature (Figures 6(b)–6(d)) [22, 41]. Both the compounds **1** and **2** displayed inhibition of AChE (IC_50_; 32.697 ± 0.340 and 29.06 ± 0.453 *μ*g/ml) and BChE (IC_50_; 17.510 ± 0.101 and 22.767 ± 0.162 *μ*g/ml) enzymes as well as antioxidant activity (IC_50_ for DPPH scavenging: 3.477 ± 0.084 and 2.580 ± 0.038 *μ*g/ml) (Figure 7). Comparison of activities revealed the compound **2** to be more potent than the compound **1** in respect of acetylcholinesterase inhibition and antioxidant activity. Additional works are warranted for isolation and evaluation of the remaining active compounds in the EAF-PP.

## 4. Discussion

Discovering new drugs for AD is a major challenge of the present moment. Extensive research on AD has elucidated numerous mechanisms of pathogenesis that offered the targets for development of therapeutics [54]. Among the targets, cholinesterase has attracted much interest due to the best therapeutic outcome. In addition, oxidative stress, which is associated with the progression of AD, has been emerged as another target to prevent and halt the progression of AD [55]. The multifactorial nature of AD necessitates the development of drug that will target both the cholinesterases and oxidative stress. Polyphenolics from natural products have received intense interest not only due to their safety and antioxidant activity, but also for other biological activities including inhibition of cholinesterase [56, 57]. Individual polyphenolic components such as resveratrol, curcumin, catechin, quercetin, and luteolin, as well as the polyphenolic mixtures from grape seed and lotus, have been reported to exert protective effects against oxidative stress-induced damage and cholinergic dysfunction in AD [20]. *Loranthus globosus* (Bengali name—Dhara), is a Bangladeshi mistletoe that grows on the host mango (*Mangifera indica*) tree and distributed throughout the country. The plant has been indicated in traditional medicine for different disorders [16, 17]. In this study, we report that *L. globosus* is a rich source of polyphenolics and possesses potential antioxidant and cholinesterase inhibitory activity.

Medicinal plants exhibit the biological activity due to presence of diverse secondary metabolites. Polyphenols are a major class of natural products that can be exploited as neuroprotective agents. These compounds contain hydroxyl groups that have the ability to display the antioxidant activity [58, 59]. Polyphenols exist in the plant as nonflavonoids such as phenolic acids and as flavonoids such as flavones. More often, catechin has been found to occur in the plant in association with epicatechin, which is known as proanthocyanidin [60]. In this study, we carried out a quantitative analysis of polyphenolics in the crude extract and fractions from *L. globosus*. We found a high content of phenolics, flavonoids, and proanthocyanidin in the crude methanolic extract (CME) (Table 1). Among the fractions of CME, the highest content appeared to be present in the EAF followed by AQF, CLF, and PEF. Several reports have shown earlier the polyphenolic constituents and the antioxidant activity of the different species of the genus *Loranthus* [21–27]. These results report for the first time that *L. globosus* has large amount of polyphenolics that might exert potential antioxidant activity.

Accumulation of evidences implicates oxidative stress in the neurotoxicity of AD [5, 6]. Oxidative stress resulting from excessive generation of A*β*-induced free radicals in neuronal cell can cause serious damage to the cell and cell death [7, 8]. Antioxidants from plant sources that counteract the free radicals by scavenging have shown effectiveness in the reduction of oxidative stress as well as oxidative stress-induced cell damage and death [9]. In this work, we evaluated the antioxidant activity of the CME and its fractions in several *in vitro* assays/models. The radical scavenging assay using DPPH is a rapid method for evaluation of the antioxidant activity. Our results demonstrated a strong radical scavenging activity of the CME (IC_50_, 4.156 ± 0.088 *μ*g/ml). Although the activity was found to be distributed in all the fractions of the CME, EAF showed the highest activity (IC_50_, 3.130 ± 0.022 *μ*g/ml) which was even greater than that of the standard antioxidant catechin (IC_50_, 3.41 ± 0.004 *μ*g/ml) used in this study (Figure 1(a)). Among the free radicals produced in the biological system, hydroxyl radical is the most reactive that affects almost all biomolecules of the cell. Our results revealed the hydroxyl radical scavenging activity of the CME and its fractions. Similar to DPPH radical scavenging, the CME and its fraction EAF exhibited good activity with IC_50_ of 15.600 ± 0.375 and 12.623 ± 0.268 *μ*g/ml, respectively (Figure 1(b)). Likewise, the CME and its fractions were found to display the hydrogen and proton donating abilities as revealed from the reducing power and total antioxidant activity assays. A marked activity was observed in the EAF which was higher than of the standard antioxidant catechin used as positive control (Figures 1(c) and 1(d)). Free radicals are well known to attack on lipids in neuronal membrane resulting in lipid peroxidation. It is extensive in AD and considered as a biomarker of oxidative stress [6, 8]. The results (Figure 2) demonstrated the potential of CME and its fractions toward the reduction of lipid peroxidation. EAF exhibited the highest activity among the extract and fractions with an IC_50_ value of 25.997 ± 0.246 *μ*g/ml. The antioxidative property of the EAF had considerably high when compared with the other species of Loranthus [21, 24, 26]. Taken together, EAF possesses potential antioxidant activity which might be effective in the prevention of oxidative damage in AD.

Cholinesterase inhibitors are used as the first line pharmacotherapeutics in AD. Cholinesterase inhibitors can elevate the level and activity of acetylcholine in the brain and improve memory and cognition [12]. Plant and plant-derived phytochemicals, which are used as alternative medicine in AD, have cholinesterase inhibitory properties [61, 62]. In this study, the CME of *L. globosus* exerted inhibition of AChE and BChE enzymes with the IC_50_ values of 153.767 ± 2.409 and 155.733 ± 0.907 *μ*g/ml, respectively, which was comparable to that of the other medicinal plants used in traditional medical systems including Ayurvedic and Unani (Figure 3) [63, 64]. Further, among the fractions of CME, we observed a marked inhibition in the EAF followed by AQF with IC_50_ of 64.987 ± 0.669 and 87.417 ± 0.61 *μ*g/ml for AChE and 85.27 ± 0.982 and 129.267 ± 1.002 *μ*g/ml for BChE, respectively. These results suggested that the extract and fractions of *L. globosus* have dual antioxidant and cholinesterase inhibitory activities.

Polyphenols are well known for its association with the antioxidant activity [65, 66]. In preliminary analysis, we observed that the antioxidant and cholinesterase inhibitory activity was high in the polyphenol rich fractions. Therefore, to explore the association of the polyphenolics with antioxidant and cholinesterase inhibitory activities, a detailed correlation studies were performed by Pearson's test. The results showed a significant association of phenolics, flavonoids, and proanthocyanidin with antioxidant and cholinesterase inhibitory activities (Table 2). To ascertain the role of polyphenols in the activity, we have isolated the polyphenolics from EAF and AQF (termed as EAF-PP and AQF-PP) separately by column chromatography using diaion resin and evaluated their activities similarly. With the increase of polyphenols, a significant increase of antioxidant property and cholinesterase inhibitory activities of the EAF-PP and AQF-PP was observed, suggesting further the role of polyphenolics in the bioactivity (Figure 4). EAF-PP was found to be more potent than AQF-polyphenolics in respect of both antioxidant and cholinesterase inhibitory activities. Kinetic studies of EAF-PP revealed a noncompetitive antagonism against both the acetylcholinesterase and butyrylcholinesterase (Figure 5).

To gain insights into the compounds responsible for bioactivity, we performed LC-MS analysis of the EAF-PP, which tentatively identified 36 compounds (Table 3). These compounds fall into two major categories, nonflavonoids such as phenolic acids and flavonoids such as flavonols, flavones, flavonols, flavonones, and proanthocyanidin. Gallic acid and its derivatives, p-coumaric acid and its derivatives, quinic acid, ferulic acid, quinic acid, rosamarinic acid, coniferin, and linamarin gallate were the important phenolic acids detected in the EAF-PP. The occurrence of these phenolic acids is common in fruits and vegetables, and the antioxidant activity of these compounds is well established [43–54, 67, 68]. The flavonoid catechin has been found to be the major constituent of tea, grapes, lotus, and other fruits [67–69]. In these plants, catechin also occurs in combination with gallic acid such as catechin gallate, gallocatechin, and gallocatechin gallate. In addition, few other common flavonoids such as quercetin, kaempferol, apigenin, and naringenin also occur in these plants. Interestingly, all these flavonoids and their derivatives have been found in the EAF-PP. Extensive information on the antioxidant and acetylcholinesterase potential of these compounds has been documented, which suggests that EAF-PP may be used in the treatment of AD. Recently, Okello et al. [70] reported that catechin, epicatechin, and epicatechin gallate, while in a mixture, exhibited synergistic activity in terms of inhibition of cholinesterases. This can explain, in part, the strong cholinesterase inhibitory activity of the EAF-PP.

Proanthocyanidins are the oligomeric and polymeric compounds formed from catechin and epicatechin and abundant in grapes, lotus, mango, and many other fruits [60]. Proanthocyanidins have received much attention due to their potential neuroprotective properties. Proanthocyanidins from grape seed and lotus have shown significant improvement in memory and cognition through multiple mechanisms including modulating oxidative stress and cholinergic neuron function [60, 71]. In this study, we have detected proanthocyanidin molecules catechin dimer and catechin tetramer glucose in EAF-PP by LC/MS. Through bioactivity-guided approach, the two major compounds catechin and catechin dimer have been isolated and characterized from EAF-PP (Figure 6). In an earlier investigation, we reported the isolation of catechin from the same ethylacetate fraction of this plant [18] and Wong et al. characterized a catechin trimer in *Loranthus parasiticus* [23]. The antioxidant activity of catechin and its polymer have been studied extensively and showed that the activity is proportional with the degree of polymerization [72], which is consistent with our result (Figure 7). In the current study, we show that catechin dimer has cholinesterase inhibitory activity which shows high specificity for acetylcholinesterase than that of catechin. So far, this report appears to be the first describing the isolation of catechin and catechin dimer as the active molecules relevant to AD treatment from this species. More studies are warranted for isolation and evaluation of the remaining active compounds in the EAF-PP.

## 5. Conclusions

This study revealed the antioxidant and cholinesterase inhibitory activity of the crude methanolic extract of *L. globosus*. The crude extract was found to contain a good amount of polyphenolics. When fractionated by solvents of different polarity, highest activity and polyphenolic content were observed in the EAF amongst the fractions. Correlation analysis showed a significant association of polyphenolic content with the antioxidant and cholinesterase inhibitory activities. The polyphenolics (EAF-PP) were isolated from the EAF that exhibited the most potent antioxidant and cholinesterase inhibitory activity. Thirty-six polyphenolic compounds were tentatively identified in the EAF-PP which were reported to have antioxidant and neuroprotective activity. Finally, two compounds catechin and catechin dimer were identified in the EAF-PP as the major active compounds. Hence, EAF-PP represents a source of potential antioxidants and cholinesterase inhibitors that may be used in the prevention and treatment of AD. Additional research in animal model of AD will be required to justify the therapeutic potential of EAF-PP.

## Figures and Tables

**Table 1:** Total phenolic, total flavonoid, and total proanthocyanidin contents of the CME and its fractions from *Loranthus globosus.*

Sample	TPC mg GAE/gm dried sample	TFC mg CE/gm dried sample	TPAC mg CE/gm dried sample
CME	336.989 ± 1.837	180.000 ± 2.060	291.0 ± 1.500
PEF	64.803 ± 0.448	49.714 ± 1.512	57.0 ± 1.500
CLF	30.753 ± 1.55	5.267 ± 0.162	21.0 ± 1.500
EAF	270.466 ± 0.657	281.715 ± 2.060	278.0 ± 2.291
AQF	240.932 ± 1.510	60.191 ± 3.805	179.0 ± 0.866

CME: crude methanolic extract; PEF: petroleum ether fraction; CLF: chloroform fraction; EAF: ethylacetate fraction; AQF: aqueous fraction; TPC: total phenolic content; TFC: total flavonoid content; TPAC: total proanthocyanidin content; GAE: gallic acid equivalent; CE: catechin equivalent.

**Table 2:** Correlation of total phenolic, total flavonoid, and total proanthocyanidin contents with antioxidant and cholinesterase inhibition activities.

Assays	Correlation coefficient (*R*^2^) values		
	TPC	TFC	TPAC
DPPH radical scavenging	0.7689	0.6021	0.6612
Lipid peroxidation inhibitions	0.6976	0.6930	0.6810
Acetylcholinesterase inhibition	0.4389	0.6274	0.5528
Butyrylcholinesterase inhibition	0.9056	0.7410	0.8274
Hydroxyl radical scavenging	0.8692	0.8771	0.9015
Reducing power assay	0.6179	0.8810	0.5680
Total antioxidant activity	0.7653	0.9267	0.8665

TPC: total phenolic content; TFC: total flavonoid content; TPAC: total proanthocyanidin content.

**Table 3:** LC/MS analysis of the EAF-PP.

Sl no.	Proposed compounds	Mode of ionization	Observed mass (*m*/*z*)	References
*Phenolic acids*				
1	Gallic acid	[M-H]^+^	171.0	[41–43]
2	Ethyl gallate	[M-H]^+^	221.1	[41, 42]
3	Gallic acid 3-*O*-gallate	[M-H]^+^	322.5	[43]
4	Galloyl glucose	[M-H]^+^	333.0	[43]
5	Octa-*O*-galloyl glucose	[M-H]^+^	1396.5	[44]
6	Quinic acid	[M-H]^+^	190.7	[42]
7	Ferulic acid	[M-H]^+^	195.1	[42]
8	*p*-Coumaric acid	[M-H]^+^	165.0	[42]
9	*p*-Coumaroyl tartaric acid	[M-H]^+^	297.5	[43]
10	*p*-Coumaroyl-4-O-glucoside	[M-H]^+^	326.4	[43]
11	3-*p*-Coumaroylquinic acid	[M-H]^+^	339.1	[43]
12	Coniferin	[M-H]^+^	340.6	[42]
13	*p*-Amino benzoate	[M-H]^+^	137.7	[42, 43]
14	Syringetin-7-*O*-hexoside	[M-H]^+^	509.6	[42]
15	Rosamarinic acid	[M-H]^+^	359.2	[45]
16	Sinapic acid hexoside	[M-H]^+^	384.9	[46]
17	Linamarin gallate	[M-Na]^+^	421.3	[22]
*Flavonoids*				
18	Catechin	[M-Na]^+^	311.3	[47]
19	(+) Catechin 3-*O*-gallate	[M-H]^+^	441.2	[42, 43]
20	(+) Epicatechin 3,5,7-trigallate	[M-H]^+^	747.2	[48]
21	Epigallocatechin-3-*O*-gallate	[M-H]^+^	459.8	[42, 49]
22	Epigallocatechin dimer	[M-Na]^+^	627.5	[49]
23	*O*-methylated(+) catechin gallate	[M-H]^+^	456.7	[50]
24	Kaemferol	[M-Na]^+^	310.4	[47]
25	Kaemferol-3-*O*-acetylglucoside	[M-H]^+^	488.5	[42]
26	Kaemferol-7-*O*-rhamnoside	[M-H]^+^	434.8	[42]
27	Quercetin	[M-Na]^+^	325.05	[47]
28	3,7-Di-O-methyl quercetin	[M-H]^+^	329.0	[42]
29	Rhamnetin	[M-H]^+^	317.0	[43]
30	Apigenin	[M-H]^+^	270.6	[42]
31	Apigenin-7-O-glucoside	[M-H]^+^	433.9	[51]
32	Naringenin	[M-H]^+^	272.9	[42, 46]
33	Naringin-7-*O*-glucoside	[M-H]^+^	434.8	[42]
34	Myrecetin rhamnohexoside	[M-H]^+^	627.6	[46]
*Proanthocyanidins*				
35	Catechin dimer	[M-H]^+^	577.3	[42, 50]
36	Catechin tertramer glucose	[M-H]^+^	1379	[47]

## Data Availability

The data presented in this study are available on request from the corresponding author.
